# Inhibitory Effects of *Chung Hun Wha Dam Tang* (CHWDT) on High-Fat Diet-Induced Obesity via AMP-Activated Protein Kinase Activation

**DOI:** 10.1155/2012/652473

**Published:** 2012-08-29

**Authors:** Md. Jamal Uddin, Yeonsoo Joe, Min Zheng, Sena Kim, Hoyoung Lee, Tae-Oh Kwon, Hun Taeg Chung

**Affiliations:** ^1^School of Biological Sciences, University of Ulsan, Ulsan 680-749, Republic of Korea; ^2^School of Medical Sciences, University of Ulsan, Ulsan 680-749, Republic of Korea; ^3^Department Medical Research, Korea Institute of Oriental Medicine 1672 Yuseongdae-ro, Yuseong-gu, Daejeon 305-811, Republic of Korea; ^4^College of Life Science and Natural Resources, Wonkwang University, Iksan 750-749, Republic of Korea

## Abstract

The *Chung Hun Wha Dam Tang* (CHWDT) herbal combination was reported to cease dizziness and phlegm. However, the effect of CHWDT in obesity has not yet been known mechanically. Therefore, we investigated whether this CHWDT could protect the cells from lipogenesis, gluconeogenesis, and inflammation in both in vivo and in vitro. CHWDT significantly decreased body weight, epididymal and perirenal fat content without affecting feed intake in high-fat diet-induced obese mice model. Additionally, CHWDT inhibited obesity-induced SREBP1, FAS, PGC1**α**, G6Pase, PEPCK and increased CPT1, ACO, and LCAD genes expression in vivo and in vitro. Proinflammatory cytokines like TNF-**α** and iNOS expression were reduced by CHWDT in both Raw264.7 macrophages and HepG2 cells. In addition, NO production was also significantly decreased by CHWDT in LPS-stimulated macrophages. Furthermore, AMPK**α** activation by CHWDT was involved in inhibition of obesity by reducing triglycerides production and increasing CPT1 expression. Based on all of the results, we suggest that CHWDT has inhibitory effects on obesity-induced lipogenesis, gluconeogenesis, and inflammation via AMPK**α** activation.

## 1. Introduction

In recent years, obesity is the most common metabolic disease emerging as a global problem especially in developed nations. Obesity is closely associated with life-style-related diseases such as atherosclerosis and noninsulin-dependent diabetes mellitus and with increased risk of coronary heart disease [1]. In addition, it is characterized by the activation of an inflammatory process in metabolically active sites such as adipose tissue, liver, and immune cells [2]. Therefore, obesity is related to overexpression of gluconeogenic, lipogenic, and inflammatory genes. In gluconeogenesis, there are two rate-limiting enzymes, phosphoenolpyruvate carboxykinase (PEPCK) and glucose-6-phosphatase (G6Pase) [3]. In addition, sterol regulatory element-binding protein 1c (SREBP1c) and fatty acid synthase (FAS) are the major regulators of lipogenic genes involved with fatty acids synthesis [4]. The relation of obesity and inflammatory response is usually characterized by abnormal adipokine production and activation of some proinflammatory signaling pathways [5, 6]. Recent evidence shows that treatment with resveratrol, a polyphenolic compound enriched in grapes and red wine, ameliorates elevated levels of tumor necrosis factor (TNF)-*α*, interleukin (IL)-6, and cyclooxygenase-2 in experimental diabetic neuropathy [7]. Besides, lipolytic genes such as carnitine palmitoyltransferase I (CPT1), long chain acyl CoA dehydrogenase (LCAD), and acyl-CoA oxidase (ACO) are regulated in obese condition [8]. Also, AMP-activated protein kinase (AMPK) is well known as a master energy sensor to maintain whole body energy homeostasis [9]. Activated AMPK in the liver is involved in the regulation of fatty acid oxidation through the phosphorylation of acetyl-CoA carboxylase (ACC), subsequently leading to reduction of malonyl-CoA, an allosteric inhibitor of CPT1 [10]. Therefore, AMPK activation leads to a concomitant inhibition of fatty acid synthesis and activation of fatty acid oxidation.

Up to now, many kinds of medicine have been developed for obese and diabetic patients of which mostly are chemical agents. However, considerable side effects always come with these agents [11]. In the world, most of the traditional therapies involve the use of herbal extracts with minimum side effects. In oriental medicine, different types of herbal plant have been found to have beneficial effects on diabetes [12]. For example, Ginseng reduces hyperglycemia in the diabetic mouse model induced by streptozotocin [13]. Also, ginseng extracts made from root, rootlet, berry, and leaf of *Panax quinquefolium *(American ginseng) and *Panax ginseng *(Asian ginseng) are proved for antihyperglycemia, insulin sensitization, islet protection, antiobesity, and antioxidation in many model systems [14]. Traditional Korean herbal medicine usually composed of different compounds having various functional roles [15]. Multiherbal formula is considered to have numerous functions due to synergistic actions and preventing adverse effects. 

*Chung Hun Wha Dam Tang *(CHWDT) has been in widespread use for four hundred of years in traditional Korean medicine. It is known well that CHWDT relieves dizziness, phlegm, and inflammation [16–18]. However, the beneficial effects of CHWDT on obesity remain to be elucidated. In this study, we have showed that CHWDT exerts an antiobesity effect through downregulating lipogenesis, gluconeogenesis, and regulation of CPT1 expression via AMPK activation. Thus, we suggest that CHWDT may be used for the therapy of obesity with minimum side effects.

## 2. Methods and Materials

### 2.1. Reagents

Lipopolysaccharide (LPS), sodium palmitate, Griess reagents, MTT (3-[4,5-dimethylthiazol-2-yl]-2,5-diphenyl tetrazolium bromide), and metformin were from Sigma-Aldrich (St. Louis, MO, USA). Also, all other chemicals were obtained from Sigma-Aldrich.

### 2.2. Preparation of CHWDT

Herbal plants were purchased from Omniherb (Daegu, Korea). In our study, we prepared CHWDT with combination of 16 different types of herbal plant (supplemental Table 1 available online at doi: 10.1155/2012/652473). The mixture of herbs was subjected to freeze-drying to make powder. Further, 12.5 g from lyophilized powder of CHWDT was taken in 125 mL of distilled water and then mixed properly. 50 mL conical tubes containing sample were centrifuged at 3,000 ×g for 20 min. The extraction was repeated three times. The extracts were then filtered through a filer paper. The filtrates were collected and final concentration of the extract was calculated as 100 mg/mL. Prepared compound (CHWDT) was stored at −20°C. The HPLC profile of CHWDT was provided in supplemental Figure 1. 

### 2.3. Animals

Seven-week-old male C57BL/6 mice were obtained from ORIENT (Pusan, Korea). The mice were maintained under specific pathogen-free conditions at 18–24°C, 40–70% humidity, 12 h light/dark cycle, and access to drinking water *ad libitum*. The normal control group was being fed with standard laboratory chow and high fat diet (Research diet Inc. D12492, New Brunswick, NJ) groups were being fed with high fat diet for 7 weeks. Body weight was recorded once in a week and feed intake was recorded twice in a week. As we expected, by the end of 7 weeks period, the body weight of high-fat-diet-fed mice were approximately 20% higher than the normal control group. Then, we started oral administration of CHWDT (800 mg/kg) in every other day basis for more 7 weeks [19]. Experiments with mice were approved by the Animal Care Committee of the University of Ulsan.

### 2.4. Cell Culture

Raw264.7 and HepG2 cells were cultured in DMEM in addition with 10% fetal bovine serum (FBS) and 1% penicillin streptomycin at 37°C in 5% CO_2_ until 75–80% confluence, where AML cells were cultured in DMEM/F12 medium with 0.005 mg/mL insulin, 0.005 mg/mL transferring, 5 ng/mL selenium, 40 ng/mL dexamethasone, and 10%FBS (ATCC CRL-2254). 

### 2.5. MTT Assay

Cell viability was checked by MTT (3-[4, 5-dimethylthiazol-2-yl]-2, 5-diphenyl tetrazolium bromide) assay. HepG2 cells were seeded in 96-well plate at a concentration of 1 × 10^4^ cells/well. After one day, the old medium was discarded and fresh medium containing 50, 100, and 200 *μ*g/mL of CHWDT were used. Twenty-four hours later, the medium was replaced with medium coupled 1 mg/mL of MTT (Sigma) as per the manufacturer's instructions. Samples were incubated with MTT reagent for 4 h at 37°C. After 4 h, samples were detected at 570 nm with an ELISA plate reader using spectrophotometer. The wavelength at 690 nm was deducted from the 570 nm.

### 2.6. Determination of Intracellular Lipid Content

As indicator of intracellular lipid content, triglycerides (TG) detection was performed according to TG-S kit (AM 157S-K, TG-S, ASIAN PHAR. Co. Ltd. Korea). In brief, after 24 h incubation 35 *μ*L from ethanol and 30% KOH mixture was added to harvested cell lysates and allowed to incubate for 3 h at 55°C. Then 65 *μ*L of 50% ethanol was added to each sample. After vortexing, samples were centrifuged for 5 min at 13000 rpm. 100 *μ*L of supernatant was separated to new tubes and again 20 *μ*L of 50% ethanol was added to each tube. From total volume of 120 *μ*L, 20 *μ*L was taken in new tube and 21.5 *μ*L of 1 M MgCl_2_ was added. After vortexing, samples were kept at 4°C for 10 min and spun down. Finally, 1 *μ*L from each samples were incubated for 10 min with 150 *μ*L TG-S reagents in 96-well plate and samples were detected at 550 nm absorbance by spectrophotometer. TG content was calculated as mg/dL.

### 2.7. Western Blotting

Cell extracts were prepared by using lysis buffer containing RIPA buffer, protease inhibitor, and phosphatase inhibitors. Protein concentration in the cells was measured by BCA assay (Pierce Biotechnology Inc., Rockford, IL, USA). An equal amount of proteins for all samples were subjected to electrophoresis and proteins were transferred to polyvinylidene difluoride (PVDF) membrane. The membranes were blocked with 5% nonfat milk in PBS containing 0.1% Tween 20 (PBS-T) for 20 min and incubated at 4°C overnight with primary antibodies and followed by secondary antibodies conjugated with horseradish peroxidase for p-AMPK, AMPK, PGC1*α*, NOS2 (iNOS, inducible nitric oxide synthase), and beta-actin. The bands were visualized by using enhanced chemiluminescence Western blotting detection system (GE Healthcare Life Sciences, Buckinghamshire, UK). Anti-p-AMPK, AMPK, PGC1*α* antibodies were purchased from Cell Signaling Technology, Inc. (Danvers, MA). Anti-NOS2 (iNOS, inducible nitric oxide synthase), *β*-actin, and anti-goat antibody conjugated to horseradish peroxidase were obtained from Santa Cruz Biotechnology (Santa Cruz, CA).

### 2.8. Reverse Transcription-Polymerase Chain Reaction (RT-PCR)

Total RNA was extracted using TRIzol reagent (Invitrogen, CA, USA) according to manufacturer's instructions. In short, 2 *μ*g of total RNA was used to make cDNA by using M-MLV reverse transcriptase (Promega Corporation, WI, USA) and oligo (dT) 15 primer (Promega Corporation, WI, USA). The resulted cDNA was subjected to PCR for mouse GAPDH, 18s, SREBP1c, FAS, CPT1, ACO, LCAD, PGC1*α*, G6Pase, PEPCK, TNF*α*, and iNOS; and human GAPDH, SREB1c, FAS, CPT1, PGC1*α*, G6Pase, PEPCK, TNF*α*, and iNOS, (Sequences and Molecular weight are given in Supplemental Table 2.) PCR was performed having the following conditions: denaturation temperature 94°C for 0.5 min, annealing temperature (according to respective primer) for 0.5 min, and extension temperature 72°C for 1 min, and PCR cycle was determined according to a kinetic profile. However, GAPDH and 18s were used as internal loading control to normalize all PCR products. Band intensities of the amplified DNAs were compared after visualization on an UV transilluminator.

### 2.9. Detection of Nitric Oxide (NO) Production

Raw264.7 cells were pretreated with CHWDT for 1 h and then LPS (1 *μ*g/mL) was treated for 24 h. Nitrite production in the medium was assessed by measuring nitrite/nitrate, the stable degradation products of NO. The culture medium was incubated with coenzyme at 37°C for 15 min. Next, the reaction mixture was incubated with nitrate reductase (Sigma-Aldrich) at 37°C for 30 min. The Griess reagent (1% sulfanilamide in 5% phosphoric acid and 0.1% naphthylethylenediamine dihydrochloride in distilled water) was added to the reaction mixture. The absorbance of the mixture at 530 nm was determined using a microplate reader, and nitrite concentration was determined using a dilution of sodium nitrite as a standard [20].

### 2.10. Statistical Analysis

Data were analyzed using a one-way ANOVA followed by Tukey's multiple-comparison test to correct for multiple observations or by an unpaired *t*-test when only two groups were compared.

## 3. Results

### 3.1. Effect of CHWDT on Metabolic Parameters in High Fat Diet-Induced Obese Mice

**Figure 1:**
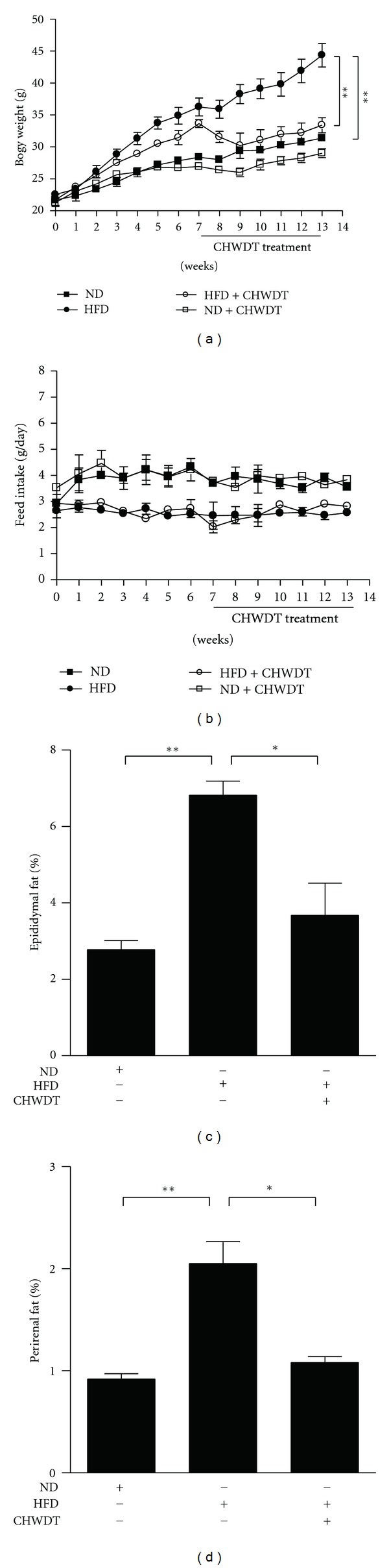
Effect of CHWDT on body weight, feed intake, and adipose fat pads in mice. CHWDT (800 mg/kg) decreased high fat diet-induced body weight (a) It had no any significant effect on feed intake (b) and decreased high fat diet-induced epididymal (c) and perirenal fat content in mice (d) There were four groups like normal diet (ND), ND + CHWDT, high fat diet (HFD), and HFD + CHWDT group. CHWDT was introduced after 7 weeks of HFD intake and mice were maintained for 14 weeks. Data were presented as *n* = 3, mean ± SEM, *P* < 0.05 as *, and *P* < 0.01 as **.

In this study, obesity was induced by feeding the normal mice with high fat diet for 7 weeks while body weight of obese mice was 20% higher than mice fed with normal diet. To examine the in vivo effects of CHWDT on high fat diet induced obesity, we orally administrated CHWDT (800 mg/kg) to mice every other day for 7 weeks. Interestingly, body weight of CHWDT administrated high-fat-diet-fed mice significantly decreased at 14 weeks (*P* < 0.01) (Figure 1(a)). Feed intake was measured twice a week, where normal diet group had a higher feed intake compared to high fat diet group, though there was not any significant difference between control and CHWDT-treated groups after 14 weeks (Figure 1(b)). Parallel to body weight change, epididymal fat pad and perirenal fat pad of obese mice had also a significant decrease with administration of CHWDT*  * (*P* < 0.05) (Figures 1(c) and 1(d)). Taken together, these results indicate that the CHWDT has antiobesity effect by reducing body weight and fat pad in diet-induced obese mice.

### 3.2. Effect of CHWDT on Cell Viability in HepG2 Cells

In order to detect the effect of CHWDT on cell viability in HepG2 cells, MTT assay was performed on 24 hours after CHWDT treatment. There was no significant effect of CHWDT at 50 and 100 *μ*g/mL on cell viability as shown in Supplemental Figure 2, though 200 *μ*g/mL had significant (*P* < 0.05) cytotoxic effect. Therefore, the CHWDT concentration was determined to be treated with 50 and 100 *μ*g/mL of concentration to further study the mechanisms of antiobesity effect of CHWDT.

### 3.3. Effect of CHWDT on Lipogenic Gene SREBP1c and FAS Expression

**Figure 2:**
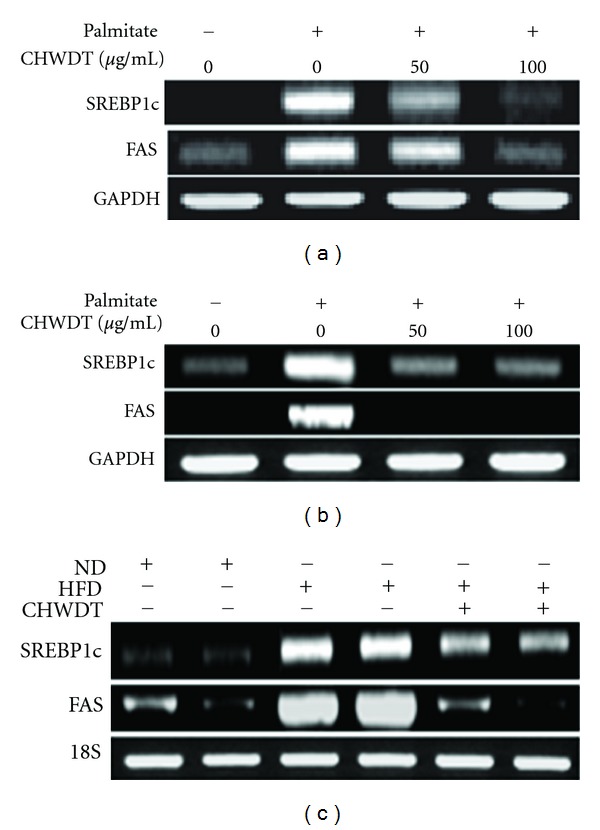
CHWDT down regulates lipogenic gene SREBP1c and FAS expression in hepatocytes. HepG2 cells (a) and AML cells (b) were pretreated with CHWDT (50 and 100 *μ*g/mL) for 1 h and palmitate (0.45 mM) was used for 12 h and mRNA levels of SREBP1c and FAS were measured. SREBP1c and FAS mRNA levels were analyzed from liver tissue collected high fat diet induced obese mice (c) The representative bands are shown from at least three independent experiments.

Obesity leads to hyperphagia, hypergluconemia, hyperlipidemia, and insulin resistance. In addition, SREBP1c is the key regulator of lipogenic genes involved with fatty acids biosynthesis like FAS [4]. In our study, HepG2 and AML cells were pretreated with CHWDT (50 and 100 *μ*g/mL) and palmitate (0.45 mM) was exposed for 12 h. Our formulated compound CHWDT significantly inhibited palmitate-induced expression of SREBP1c and FAS in both cell types (Figures 2(a) and 2(b)), suggesting a beneficial role of CHWDT on inhibition of lipogenesis. To further confirm the above results, we checked expression patterns of SREBP1c and FAS in liver tissue obtained from high fat diet-induced obese mice following administration of CHWDT. CHWDT was found to involve with suppression of SREBP1c and FAS in liver tissue in vivo (Figure 2(c)), further suggesting antiobesity effects of CHWDT.

### 3.4. Effect of CHWDT on Lipolytic Gene CPT1, ACO, and LCAD Expression

**Figure 3:**
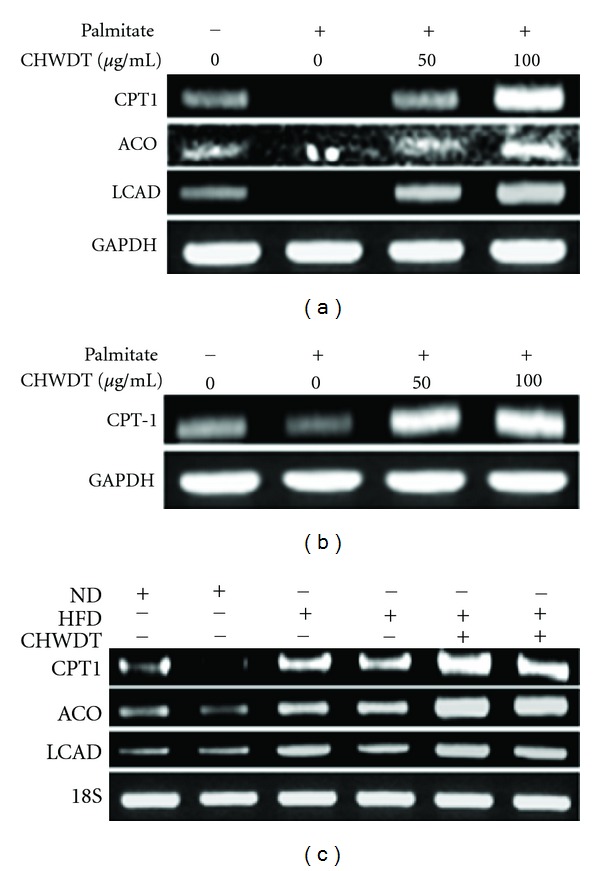
CHWDT induces lipolytic gene CPT1, ACO and LCAD expression in hepatocytes. AML cells were pretreated with CHWDT (50 and 100 *μ*g/mL) for 1 h and palmitate (0.45 mM) was used for 12 h and then CPT1, ACO and LCAD mRNA levels were observed by RT-PCR (a) HepG2 cells were pretreated with CHWDT (50 and 100 *μ*g/mL) for 1 h and palmitate (0.45 mM) was used for 12 h and then CPT1 mRNA levels were performed by RT-PCR (b) CPT1, ACO and LCAD mRNA levels were analyzed from liver tissue collected high fat diet induced obese mice (c) The representative bands are shown from at least three independent experiments.

Mitochondrial CPT1 is the main rate-limiting enzyme for fatty acids oxidation [21]. In addition, ACO, and LCAD are also involved with fatty acids oxidation [8]. However, in the present study, CHWDT was found to be associated with increased mRNA of CPT1, ACO and LCAD in AML cells (Figure 3(a)) and CPT1 in HepG2 cells (Figure 3(b)), supporting the role of herbal compound in antiobesity events. To confirm the effects of CHWDT, we analyzed CPT1, ACO, and LCAD mRNA levels in liver tissue obtained from high fat diet induced obese mice following administration of CHWDT. Consistent with in vitro results, CHWDT increased CPT1, ACO, and LCAD expression (Figure 3(c)) in liver tissue in vivo as well.

### 3.5. Effect of CHWDT on Gluconeogenic Gene PGC1*α*, G6Pase, and PEPCK Expression

**Figure 4:**
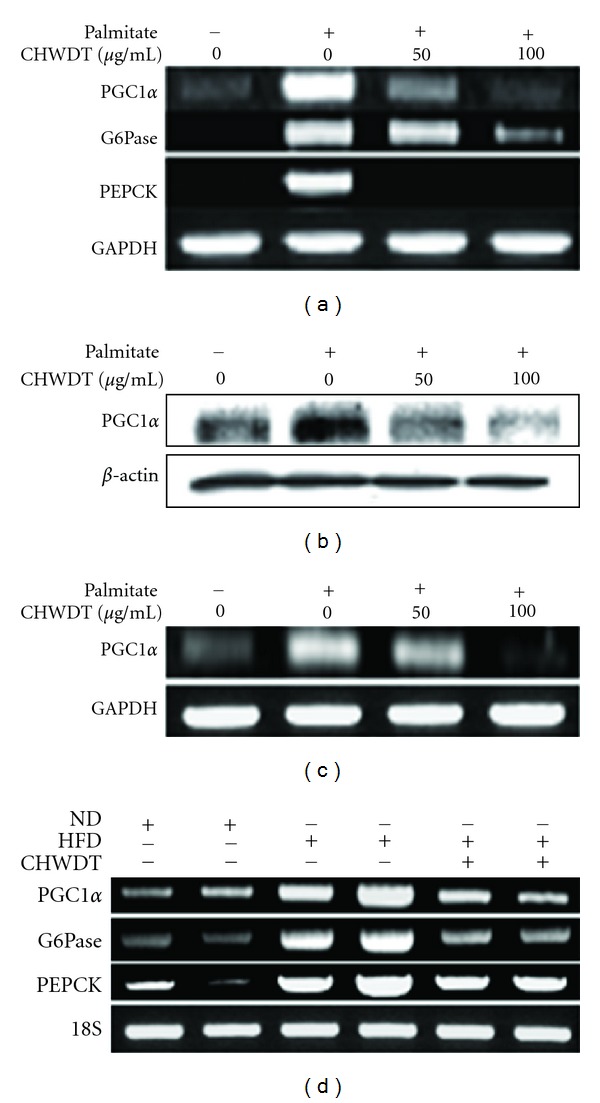
CHWDT decreases gluconeogenic gene PGC1*α*, G6Pase, and PEPCK expression in hepatocytes. PGC1*α*, G6Pase, and PEPCK mRNA levels in HepG2 cells (a) were observed. And also, representative immunoblot for PGC1*α* protein expression was shown (b) In AML cells, palmitate-induced PGC1*α* mRNA levels (c) were decreased by CHWDT treatment. After the pretreatment of 50 and 100 *μ*g/mL CHWDT for 1 h, palmitate was treated with a concentration of 0.45 mM for 24 h. PGC1*α*, G6Pase and PEPCK mRNA levels were analyzed from liver tissue collected from high fat diet-induced obese mice (d) Analysis of mRNA was done by RT-PCR. The representative bands are shown from at least three independent experiments.

PEPCK and G6Pase are target genes of PGC1*α* and are considered as important enzymes in gluconeogenesis process [22, 23]. Therefore, the effects of CHWDTon gene expression of PGC1*α* and its target genes PEPCK and G6Pase, were analyzed in HepG2 cells. Palmitate (0.45 mM) induced mRNA levels of PGC1*α*, G6Pase and PEPCK (Figure 4(a)) and protein levels of PGC1*α* (Figure 4(b)) were inhibited by CHWDT in HepG2 cells. Additionally, palmitate-induced PGC1*α* expression was reduced by pretreatment of CHWDT in AML cells as well (Figure 4(c)). To further monitor the effects of CHWDT, following administration of CHWDT liver tissue from high fat diet-induced obese mice was subjected for analysis of mRNA levels of PGC1*α*, G6Pase, and PEPCK. Similarly, CHWDT downregulated high fat diet induced PGC1*α*, G6Pase, and PEPCK expression (Figure 4(d)) in liver tissue. Thus, CHWDT was found to have a strong inhibitory effect on gluconeogenesis-related disorders.

### 3.6. Effect of CHWDT on Inflammatory Mediators iNOS, TNF-*α*, and NO Level

**Figure 5:**
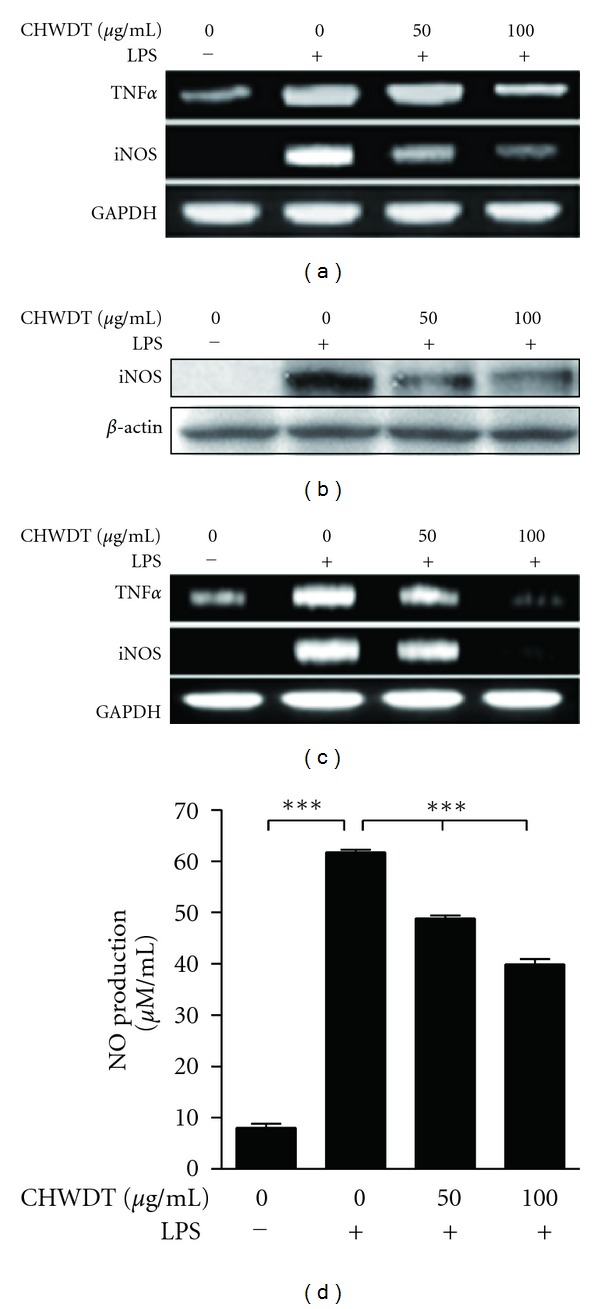
CHWDT suppresses iNOS, TNF-*α*, and NO levels. TNF-*α* and iNOS mRNA levels (a) and iNOS protein expression (b) in Raw264.7 cells and TNF-*α* and iNOS mRNA levels in HepG2 cells (c), where CHWDT (50 and 100 *μ*g/mL) was pretreated for 1 h and LPS (1 *μ*g/mL) was used for 24 h, mRNA was checked by RT-PCR and protein expression by western blotting. Data are representative of at least three independent experiments. NO production in Raw264.7 cells was observed by using Griess reagents, where CHWDT (50 and 100 *μ*g/mL) was pretreated for 1 h and LPS (1 *μ*g/mL) was used for 24 h (d) Data are presented as mean ± SEM of three independent experiments with triplicate wells and *P* < 0.001 as ***.

Obesity-induced type 2 diabetes is related to inflammation as well as increase of inflammatory markers [24]. Inhibition of such type of mediators might be a good plan to reduce obesity. Thus, to observe the effects of CHWDT on inflammatory mediators, first we pretreated the cells with CHWDT and then treated LPS (1 *μ*g/mL) for 24 h. As shown in Figures 5(a) and 5(b), iNOS and TNF-*α* mRNA and protein levels of iNOS were downregulated by CHWDT in LPS-stimulated Raw264.7 cells, respectively. Similarly, in HepG2 cells the iNOS and TNF-*α* mRNA levels (Figure 5(c)) were decreased with CHWDT. In addition, LPS-induced NO production was significantly decreased by CHWDT in Raw264.7 cells (*P* < 0.001) (Figure 5(d)). Taken together, these results suggested that CHWDT could play an important role in anti-inflammatory responses.

### 3.7. Effect of CHWDT-Induced AMPK*α* Activation on CPT1 Expression and Lipid Content

**Figure 6:**
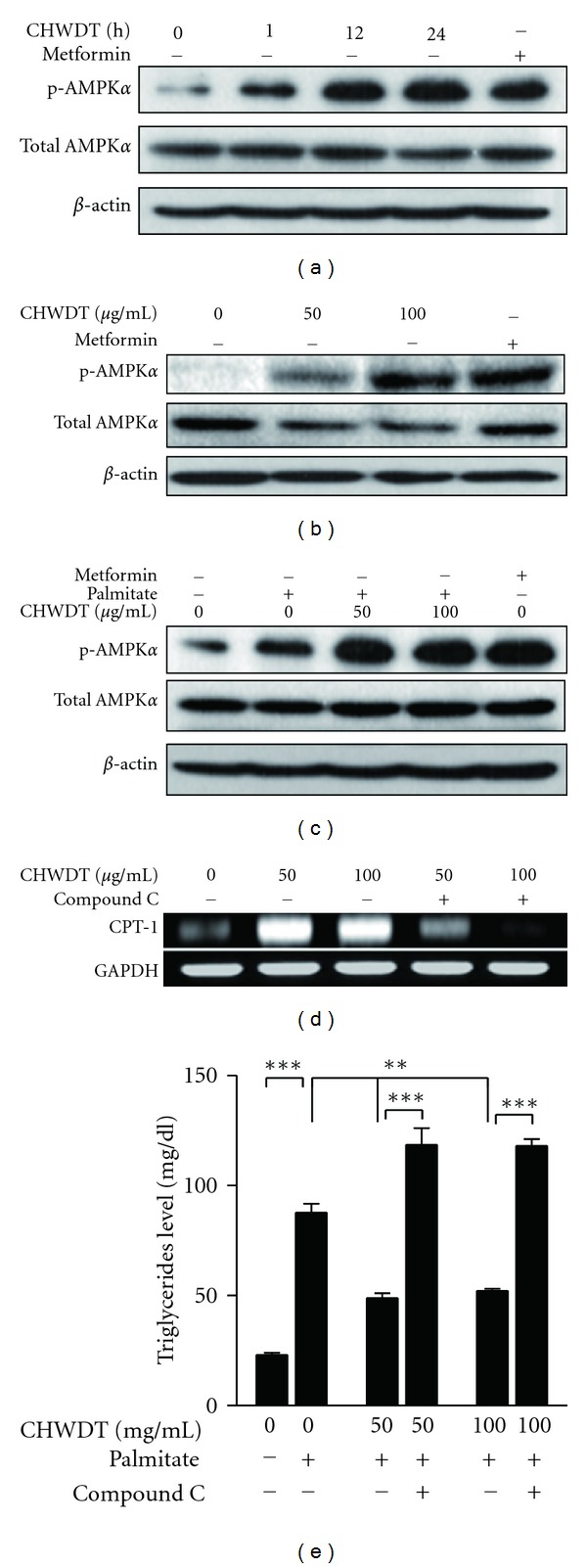
CHWDT increases CPT1 expression and reduces lipid content through AMPK*α* activation in HepG2 cells.Cells were incubated with 100 *μ*g/mL CHWDT for 1, 12, and 24 h and 2 mM metformin (a) Cells were incubated with 50 and 100 *μ*g/mL CHWDT for 9 h and 2 mM metformin (b) Cells were pretreated with CHWDT for 1 h and palmitate (0.45 mM) was treated for 12 h and 2 mM metformin (c) Cells were treated with metformin for 3 h as positive control of AMPK*α* activation. Western blotting was performed to detect phospho-AMPK*α*, total AMPK*α* and beta-actin as loading control. The representative blot is shown. Cells were pretreated with or without compound C (20 *μ*M) for 30 min and treated with CHWDT (50 and 100 *μ*g/mL) for 12 h. CPT1 mRNA was checked by RT-PCR, and representative band is shown (d) Intracellular triglycerides (TG) levels were detected by TG-S kit, where cells were treated with CHWDT (50 and 100 *μ*g/mL) in presence of palmitate (0.45 mM) for 24 h with or without preincubating with 20 *μ*M compound C for 30 min. The control cells were incubated with 1.76% bovine serum albumin (BSA) for 24 h (e) Data indicating mean ± SEM of three independent experiments with triplicate wells, *P* < 0.01 as **, and *P* < 0.001 as ***.

The key energy metabolism regulator AMPK is associated with an increase in fatty acid oxidation and AMPK activation crucial for CPT1 activity [21]. Activation of AMPK is involved with inhibition of high-fat-induced obesity through decreasing hepatic SREBP1c and FAS expression in rats [25]. In this study, *CHWDT* was applied at a concentration of 100 *μ*g/mL in a time-dependent manner (0, 1, 12, and 24 h) in HepG2 cells. Activation of AMPK*α* was found in a time-dependent manner (Figure 6(a)). Dose-dependent treatment (0, 50, and 100 *μ*g/mL for 9 h) of CHWDT also showed increase level of AMPK*α* activation in HepG2 cells (Figure 6(b)). Further, CHWDT was pretreated for 1 h and then treated with palmitate for 12 h and metformin was used as positive control of AMPK*α* activation. Interestingly, CHWDTincreased phospho-AMPK*α* expression in palmitate-treated HepG2 cells (Figure 6(c)). To investigate the mechanisms behind the antiobesity effect of CHWDT mediated through AMPK*α* activation, we used 20 *μ*M of compound C as a selective AMPK inhibitor and checked mRNA level of CPT1, a rate-limiting enzyme for mitochondrial fatty acids beta oxidation [26]. Interestingly, CHWDT-induced CPT1 expression was reduced by AMPK inhibitor (compound C) as shown in Figure 6(d) suggesting role of AMPK*α*-mediated CPT1 in antiobesity events [26]. Further to check the functional relationship between AMPK activation and obesity from lipid accumulation, we inhibited the activity of AMPK by using compound C and TG levels were checked. Consistent with previous results, CHWDT significantly decreased palmitate-induced TG levels (*P* < 0.001), whereas compound C restored palmitate-induced TG levels in HepG2 cells (Figure 6(e)) as described in [27]. Therefore, all the results indicate a role of CHWDT in lipid metabolism through at least in a part by AMPK*α*-mediated CPT1 expression.

## 4. Discussion

The history of using traditional herbal medicine is very popular all over the world due to having many beneficial health effects with minimum side effects. In our study, we tested the antiobesity effects of CHWDT with different combination of herbs. From our results, we found that the CHWDThas antilipogenic, antigluconeogenic, and anti-inflammatory effects. In addition, the inhibitory effects on lipogenesis, gluconeogenesis, and inflammation are mediated by increased expression of AMPK*α* as well as CPT1. Metabolic parameters like body weight, feed intake, and adipose fat pads are closely related to obesity leading to diabetes. We found that CHWDT improved almost all of the metabolic parameters in high fat diet induced mice compared to normal control diet. Therefore, we sought to find out the molecular mechanisms involved with antiobesity effects of CHWDT in vitro and in vivo. 

Efficient lipid metabolism is very important, because dysregulation of lipid metabolism may cause many kinds of metabolic diseases. Lipid storage in liver by lipogenesis leads to decreased hepatocytes function and causes liver diseases. In addition, influx of free fatty acids (palmitic acid and stearic acid) as a result of lipogenesis leads to increased level of lipogenic genes [28]. Upregulated SREBP1c level was revealed in NAFLD patients [29] and in livers of obese mice [30]. In contrast, increased lipogenic activity leads to decreased expression of lipolytic genes like CPT1, LCAD, and ACO [8, 21]. In the present study, we found that palmitate-induced SREBP1 and FAS expression was downregulated by CHWDT in case of HepG2 and AML cells. On the other hand, CHWDT was found to be associated with increased mRNA of CPT1, ACO, and LCAD in hepatocytes. Thus, these results support the role of herbal compound CHWDT in antiobesity events.

Gluconeogenesis is a ubiquitous process mainly takes place in human liver in which glucose is produced from noncarbohydrate carbon substrates such as lactate, glycerol, and glucogenic amino acids.The main enzymes involved in gluconeogenesis are PGC1*α*, G6Pase, and PEPCK [22, 23, 31, 32]. Though PGC1*α* is known to regulate thermogenesis, mitochondrial biogenesis, glucose uptake, and metabolism [33], PGC1*α* found to regulate glucose synthesis during gluconeogenesis [34, 35]. Therefore, inhibition of gluconeogenesis may be a good treatment strategy for obesity leading to type 2 diabetes, by inhibiting glucose production and stimulating glucose uptake by cells [36]. Different types of herbal plants are reported to reduce obesity related type 2 diabetes decreasing the level of excessive blood glucose [37]. In fact, our formulated compound CHWDT-inhibited palmitate induced gluconeogenic genes such as PGC1*α*, G6Pase, and PEPCK, ensuring noble antiobesity effects of CHWDT.

Inflammation is directly or indirectly involved with obesity as well as type 2 diabetes [24]. Inflammatory mediators, such as nitric oxide, iNOS, and TNF-*α* play important roles in the regulation of the immune system. Further, enhanced level of iNOS was found in the liver of chronic HBV-infected patients [38]. Overproduction of NO is cytotoxic and can kill pathogens directly [39] and it influences macrophages or surrounding tissues. LPS secreted from gram-negative bacteria activates TLR4 signaling pathway which triggers to secrete inflammatory cytokines and induces iNOS gene expression in immune cells leading to endotoxin shock. In the present study, LPS and palmitate-induced inflammatory mediators such as TNF-*α*, iNOS, and NO were suppressed by CHWDT in both HepG2 and Raw264.7 cells in vitro. Thus, CHWDT had anti-inflammatory potential in vitro.

It is well known that AMPK regulates variety of signaling cascades, glucose transport, hepatic lipid metabolism, downregulating fatty acid synthesis by transcription of SREBP1c [40, 41]. AMPK activation was found to regulate CPT1 activity, whereas CPT1 plays a key role in fatty acids oxidation [21]. In our study, CHWDTincreased AMPK*α* activation in a time- and dose- dependent manner. Further,*   *CHWDT-induced CPT1 expression was downregulated by compound C, indicating the role of AMPK*α* in CPT1 regulation. In addition, palmitate-induced TG accumulation was significantly reduced by CHWDT and CHWDT effect was abrogated by AMPK inhibitor, supporting role of AMPK*α* on lipid content as described in [27]. Therefore, upregulation of AMPK*α* activation by CHWDT provided evidence for functions of CPT1, ACO, and LCAD involved with fatty acids oxidation [8] as shown in Figures 3 and 6(d), connecting the role of the herbal compound in antiobesity events.

In conclusion, CHWDT had inhibitory effects on lipogenesis, gluconeogenesis, and inflammation in vitro and in vivo in high fat diet induced mice. It is suggested that antiobesity effects of CHWDT is mediated through CPT1 via AMPK*α* activation and down regulation of SREBP1, FAS, PGC1*α*, PEPCK, G6Pase, iNOS, TNF-*α*, and NO production. These findings may provide molecular evidences for the use of CHWDT as a therapy for obesity-induced disorders.

## Supplementary Material

In our study, we prepared CHWDT with combination of 16 different types of herbal plant. The mixture of herbs was subjected to freeze-drying to make powder. Further, 12.5 g from lyophilized powder of CHWDT was taken in 125 mL of distilled water and then mixed properly. 50 mL conical tubes containing sample were centrifuged at 3,000×g for 20 min. The extraction was repeated three times. The extracts were then filtered through a filer paper. The filtrates were collected and final concentration of the extract was calculated as 100 mg/mL. Prepared compound (CHWDT) was stored at *-*20°C.
