# Supplementary material for: Inhibitory Effects of Chung Hun Wha Dam Tang (CHWDT) on High-Fat Diet-Induced Obesity via AMP-Activated Protein Kinase Activation
Source: Evid Based Complement Alternat Med. 2012 Aug 29;2012:652473. doi: 10.1155/2012/652473 (PMC3437961; doi:10.1155/2012/652473)

**Supplementary Information**

**Inhibitory Effects of *Chung Hun Wha Dam Tang (CHWDT)* on High Fat Diet-Induced Obesity via AMP-activated Protein Kinase activation**

Md. Jamal Uddin^a^, Yeonsoo Joe^a^, Min Zheng ^b^, Sena Kim^a^, Tae-Oh Kwon^c^, and

Hun Taeg Chung^a^

*^a^School of Biological Sciences, ^b^School of Medical Sciences, University of Ulsan, Korea,*

*^c^College of Life Science and Natural Resources, Wonkwang University School of Medicine, Korea*

**Supplementary Table 1: Composition of *CHWDT.***

| **Name of Herbs** | **Scientific Name** | **Amount** |
| --- | --- | --- |
| Citrus Unshius Pericarpium | *Citrus unshiu* | 3.75g |
| Pinelliae Tuber | *Rhizoma pinelliae* | 3.75g |
| Poria Sclerotium | *Pachyma hoelen rumphius* | 3.75g |
| Ponciri Fructus Immaturus | *Poncirus trifoloata*. | 2.625g |
| Atractylodis Rhizoma Alba | *Atractylodes japonica* | 2.625g |
| Cnidii Rhizoma | *Cnidium officinale* | 1.875g |
| Scutellariae Radix | *Scutellaria baicalensis* | 1.875g |
| Angelica Dahuricae Radix | *Angelica dahurica* | 1.875g |
| Osterici Radix | *Curcumae Longae Rhizoma* | 1.875g |
| Ginseng Radix | *Panax ginseng* | 1.875g |
| Arisaematis Rhizoma | *Arisaematis Rhizom* | 1.875g |
| Saposhnikoviae Radix | *Ledebouriella seseloides* | 1.875g |
| Asiasari Radix et Rhizoma | *Asiasarum sieboldi* | 1.125g |
| Coptidis Rhizoma | *Coptis japonica* | 1.125g |
| Glycyrrhizae Radix | *Glycyrrhiza glabra* | 1.125g |
| Zingiberis Rhizoma Crudus | *Zingiber ojficinale* | 3 slice |

**Yield after lipholization is 16.2g (46.29%) from 35g of above mixture.*

**Supplementary Table 2: Primer sequences of gene used in this study.**

| **Primer** | **Mouse (sequences and size)** | **Human (sequences and size)** |
| --- | --- | --- |
| GAPDH | (f-aggccggtgctgagtatgtc,  r-tgcctgcttcaccttct; 530bp) | (f-ccacccatggcaaattccatggca,  r-tctagacggcaggtcaggtccacc; 520bp) |
| 18s | (f-cagtgaaactgcgaatggct,  r-tgccttccttggatgtggta; 397bp) | **-** |
| SREBP1c | (f-aaacccgaagtggtggagac,  r-cggtgtgtacccgtagcatc; 305bp) | (f-aggactgtgctctgcgagtg,  r-ggctgggtcacacagttcag; 499bp) |
| FAS | (f-ttgctgccgtgtccttctac,  r-acgcgtgatggtctgtttgt; 455bp) | (f-acggaggccatatgcttctt,  r-acgtggacggatactttccc; 335bp) |
| CPT1 | (f-gcagcattcttcgtgacgtt,  r-atgacctcctggcattctcc; 350bp) | (f-ctgagcacggcaagatgagt,  r-gtccagtttgcgcctgtaaa; 449bp) |
| ACO | (f-tggacagccaatgctggtat,  r-gctgcacgtagcttgtaggc; 329bp) | **-** |
| LCAD | (f-gaggtctgggaaaaagctgg,  r-ttcattccgttttccaccaa; 458bp) | **-** |
| PGC1α | (f-ggaactgcaggcctaactcc,  r-ttggagctgttttctggtgc; 495bp) | (f-ggaactgcaggcctaactcc,  r-cactgtccctcagttcaccg; 595bp) |
| G6Pase | (f-agactcccaggactggttca,  r-gtagaatccaagcgcgaaac; 601bp) | (f-tacgtcctcttccccatctg,  r-cctggtccagtctcacaggt; 240bp) |
| PEPCK | (f-actgttggctggctctcact, r-tgccttcggggttagttatg; 605bp) | (f-gagctgacggattcacccta,  r-ccactgccaaaggagatgat; 349bp) |
| TNFα | (f-agttctatggcccagaccct,  r-cggactccgcaaagtctaag; 463bp) | (f-aagcctgtagcccatgttgt,  r-cagatagatgggctcatacc; 365bp) |
| iNOS | (f-ccaccttggtgaagggactgagct,  r-gctgcggggagccattttggt; 381bp) | (f-cagtacgtttggcaatggagactgc,  r-ggtcacattggaggtgtagagcttg; 340bp) |

**Legend of the supplementary figure**

*Supplementary Fig. 1. CHWDT increases cell viability in HepG2 cells.*

Cells were incubated with *CHWDT* (0, 50, 100 and 200μg/ml) for 24h. After 24h, MTT assay was performed to measure the cell viability. Data indicating mean ± SEM of three independent experiments with triplicate wells, p< 0.05 as *.


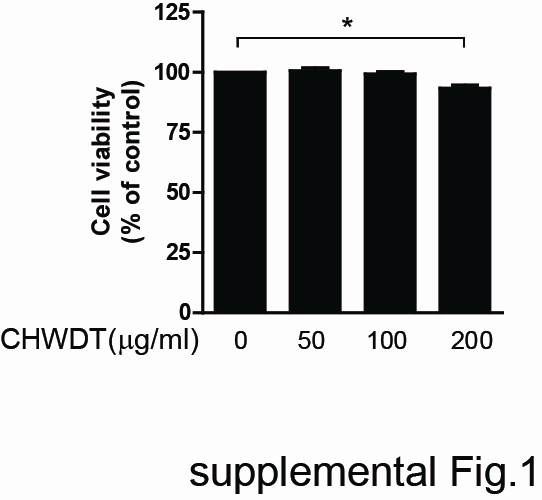

Supplement: Supplementary file 1 — In our study, we prepared CHWDT with combination of 16 different types of herbal plant. The mixture of herbs was subjected to freeze-drying to make powder. Further, 12.5 g from lyophilized powder of CHWDT was taken in 125 mL of distilled water and then mixed properly. 50 mL conical tubes containing sample were centrifuged at 3,000×g for 20 min. The extraction was repeated three times. The extracts were then filtered through a filer paper. The filtrates were collected and final concentration of the extract was calculated as 100 mg/mL. Prepared compound (CHWDT) was stored at -20°C. [file 652473.f1.doc]
